# Constructing a molecular subtype model of colon cancer using machine learning 

**DOI:** 10.3389/fphar.2022.1008207

**Published:** 2022-09-16

**Authors:** Bo Zhou, Jiazi Yu, Xingchen Cai, Shugeng Wu

**Affiliations:** ^1^ Department of General Surgery, Ningbo Medical Center Lihuili Hospital, Ningbo University, Ningbo, China; ^2^ Medical School, Ningbo University, Ningbo, China

**Keywords:** colon cancer, machine learning, molecular subtype model, pathogenesis, prognosis

## Abstract

**Background:** Colon cancer (CRC) is one of the malignant tumors with a high incidence in the world. Many previous studies on CRC have focused on clinical research. With the in-depth study of CRC, the role of molecular mechanisms in CRC has become increasingly important. Currently, machine learning is widely used in medicine. By combining machine learning with molecular mechanisms, we can better understand CRC’s pathogenesis and develop new treatments for it.

**Methods and materials:** We used the R language to construct molecular subtypes of colon cancer and subsequently explored prognostic genes with GEPIA2. Enrichment analysis is used by WebGestalt to obtain differential genes. Protein–protein interaction networks of differential genes were constructed using the STRING database and the Cytoscape tool. TIMER2.0 and TISIDB databases were used to investigate the correlation of these genes with immune-infiltrating cells and immune targets. The cBioportal database was used to explore genomic alterations.

**Results:** In our study, the molecular prognostic model of CRC was constructed to study the prognostic factors of CRC, and finally, it was found that Charcot–Leyden crystal galectin (CLC), zymogen granule protein 16 (ZG16), leucine-rich repeat-containing protein 26 (LRRC26), intelectin 1 (ITLN1), UDP-GlcNAc: betaGal beta-1,3-N-acetylglucosaminyltransferase 6 (B3GNT6), chloride channel accessory 1 (CLCA1), growth factor independent 1 transcriptional repressor (GFI1), aquaporin 8 (AQP8), HEPACAM family member 2 (HEPACAM2), and UDP glucuronosyltransferase family 2 member B15 (UGT2B15) were correlated with the subtype model of CRC prognosis. Enrichment analysis shows that differential genes were mainly associated with immune-inflammatory pathways. GFI1 and CLC were associated with immune cells, immunoinhibitors, and immunostimulator. Genomic analysis shows that there were no significant changes in differential genes.

**Conclusion:** By constructing molecular subtypes of colon cancer, we discovered new colon cancer prognostic markers, which can provide direction for new treatments in the future.

## Introduction

CRC is the most common digestive system tumors in the world. In the USA, the incidence of colon adenocarcinoma is roughly equal between men and women, and is expected to increase by 100,000 new cases and 50,000 deaths in 2022 (Siegel et al., 2022). In China, where the incidence is slightly higher in men than in women, an estimated 590,000 new cases and 300,000 new deaths are expected (Xia et al., 2022). With the development of detection technology, the early screening of CRC plays an increasingly important role. For example, colonoscopy is the most commonly used detection method. In addition, due to the development of treatment, including surgical treatment and neoadjuvant therapy, the 5-year survival rate of CRC is close to 64% (Miller et al., 2019). Although the development of new technologies has brought treatment progress in the CRC, the carcinogenesis is still unclear. Tumor development is influenced by the tumor microenvironment (TME), which contains a variety of cell types, including immune-infiltrating cells and cancer-associated fibroblasts. These cells can detach from the original normal growth and play an important role in tumor cell proliferation, differentiation, invasion, and metastasis (Schmitt and Greten, 2021). Except that the pathogenic mechanism for CRC is not clear, the classification of CRC is still mainly based on TNM staging, and this classification has insufficient understanding of CRC. Therefore, it contributes to the diagnosis and treatment of CRC by elucidating the molecular mechanism of colon carcinogenesis. In recent years, machine learning (ML)-based methods for understanding tumors have received increasing attention (Liu et al., 2020; Collins et al., 2021; Masud et al., 2021), and many algorithms for predicting and classifying tumors have emerged (Aziz et al., 2021; Karhade et al., 2021; Tohka and van Gils, 2021). Existing machine learning algorithms include linear regression, logistic regression, decision trees, support vector machines (SVM), naive Bayes, K-mean clustering method, random forest, dimensionality reduction algorithms, gradient boosts, and AdaBoost. Jiang et al. used convolution neural networks to predict the prognosis of stage III CRC (Jiang et al., 2020). Previous cases of applying machine learning have achieved good results and demonstrated strong practicality. In the present study, we construct a CRC prognostic model based on machine learning and public databases to find new prognostic markers and their relationship with CRC.

## Materials and methods

### Data source

RNA-seq data and clinical data are from the TCGA database, and these data are downloaded from the University of California Santa Cruz (UCSC) Xena database.

### Cluster analysis

We used consensus clustering analysis to randomly select 5,000 genes in the CRC samples from the TCGA database to construct molecular subtypes of CRC. The key parameters include 80% resampling, k-estimated maximum value of 6,500 repetitions, and PAC measure (PAC measure (proportion of ambiguous clustering) explained; optimal k is the k with the lowest PAC value) to filter the best k value. Prognosis between different clusters is compared using Kaplan–Meier analysis. All these analyses are performed using R package “ConsensusClusterPlus” (Wilkerson and Hayes, 2010). The clustering results will be presented in the heatmaps, and the survival analysis results will be presented by GraphPad prism7.

### Differential expression genes analysis

We performed the clusters of prognostic value for RNA-seq differential gene analysis using the R package “limma”. We performed RNA-seq data differential analysis on cluster1, which consisted of 144 samples, and cluster3 which consisted of 150 samples. To exclude the influence of extreme values or outliers, we deleted genes with no expression significance (including *p* > 0.05 or FDR>0.05). Finally, we screened the genes with |logFC|≥1 as the differential genes of cluster1 and cluster3 of the CRC subgroup.

### Survival analysis

In order to explore whether the expression levels of differential genes between the two clusters have an impact on prognosis, we used an external database to analyze the differentially expressed genes. Gene expression profiling interactive analysis (Tang et al., 2019) (GEPIA2, http://gepia2.cancer-pku.cn, version 2) is an online tool that searches the TCGA database, which collected RNA sequencing data of 9,736 tumors and 8,587 normal samples in total. The GEPIA2 database was used to analyze the effect of two clusters of differentially expressed genes on survival.

### Enrichment analysis

A web-based gene set analysis toolkit (Liao et al., 2019) (WebGestalt, http://www.webgestalt.org/option.php) can enrich genes of interest to understand their functions and pathways involved. GO analysis is a common annotation method for genes and gene products, including molecular functions, biological pathways, and cellular components. KEGG analysis is a resource for analyzing gene functions and information. In order to study the differentially expressed genes’ enrichment information of cluster1 and cluster3, we used the WebGestalt website to conduct GO and KEGG online enrichment analyses; parameters considered analytically meaningful for enrichment analysis included *p* < 0.05 and FDR<0.05.

### Protein–protein interaction analysis

We used the STRING database (https://string-db.org/) (Szklarczyk et al., 2021; Siegel et al., 2022) to explore the interaction between the proteins expressed by these genes. Through the PPI network, we could study whether these genes played a role in the prognosis of subtype models of CRC, independently or together. Then, we used the MCODE plugin of the Cytoscape software to find the core network of PPI.

### Immune infiltration analysis

In order to study the impact of these differential genes on immune function between the two clusters, we used the TIMER2.0 (Li et al., 2020) database for analysis. TIMER2.0 (http://timer.comp-genomics.org/) is a database that comprehensively analyzes the correlation between tumors and immune infiltrating cells. In addition to the TIMER2.0 database, we also used the TISIDB database to analyze the relationship of these DEGs with immunoinhibitors and immunostimulators. TISIDB (Ru et al., 2019) (http://cis.hku.hk/TISIDB/) is an online database for immune infiltration analysis based on the TCGA database.

### Genomic alteration analysis

The cBio Cancer Genomics Portal (Wu et al., 2019) (cBioportal, http://cbioportal.org) is a database that collects multiple tumor genomics. We used this tool to analyze genomic alterations in 10 genes with prognostic significance in subtypes of CRC to explore their impact.

## Results

### Machine learning divides CRC into different subtypes

**FIGURE 1:**
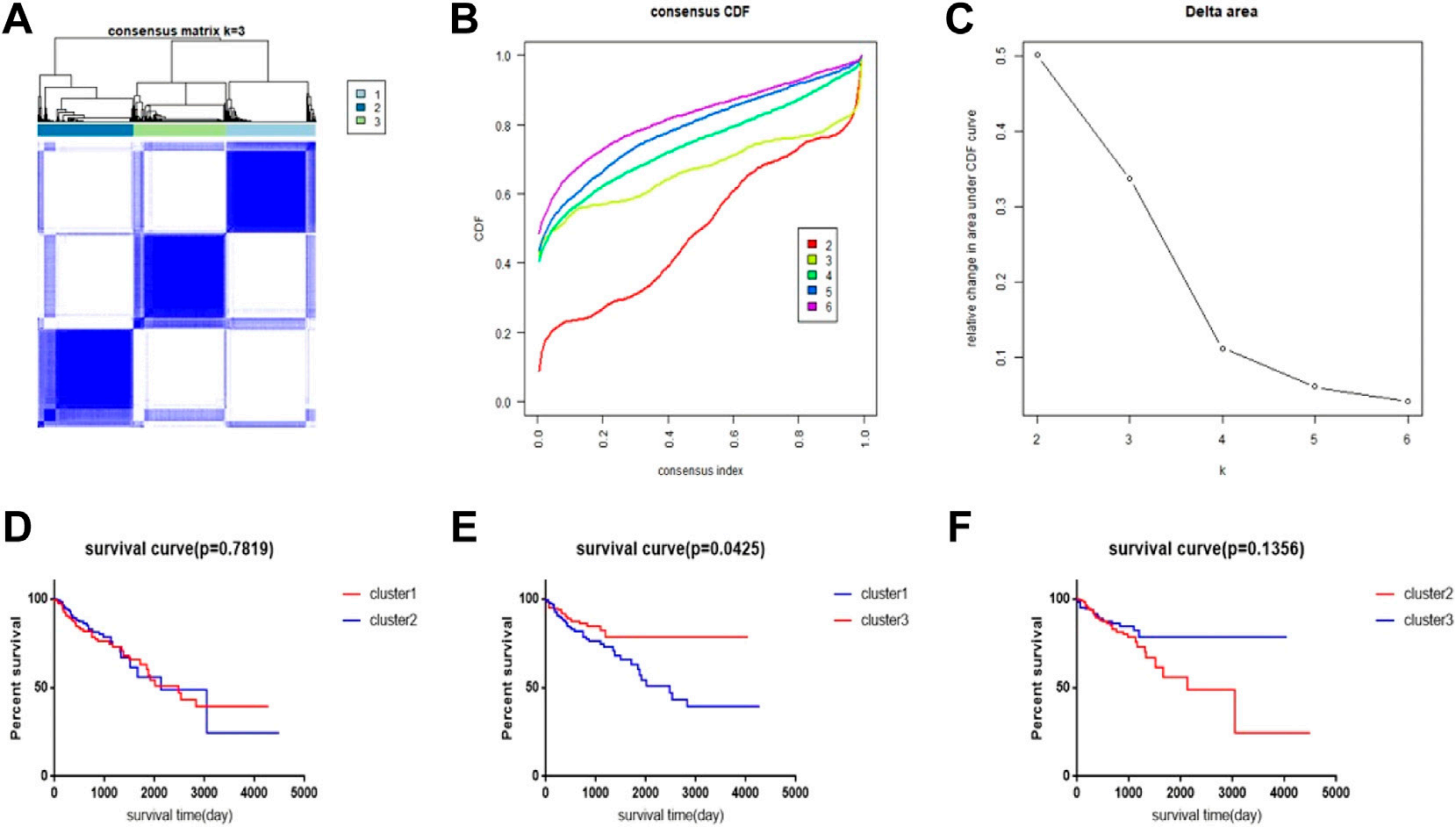
CRC subtype characteristics and difference in overall survival (OS). **(A)** Consensus clustering analysis yields three matrices, each representing one CRC subtype. **(B)** Consensus clustering cumulative distribution function (CDF) for *k* = 2–9. **(C)** Relative change in area under the CDF curve for *k* = 2–6. **(D–F)** Survival time curve between 3 clusters.

We performed a consensus clustering method of CRC samples in the TCGA database using the PAC measure to select the best value of *k* = 2 (Figures 1A–C). In the TCGA database, 448 samples with complete follow-up information were included in the study. The 448 samples were divided into three subtypes; cluster1 included 144 samples, cluster2 included 154 samples, and cluster3 included 150 samples. The rest of the clustering results are shown in Supplementary Figure S1. By comparing the survival times of the three clusters, we found a significant survival difference between clusters 1 and 3. The survival time of cluster3 was better than that of cluster1, while there was no significant difference in survival time between clusters 1 and 2 and between clusters 2 and 3 (Figures 1D–F).

### Screening of DEGs

**FIGURE 2:**
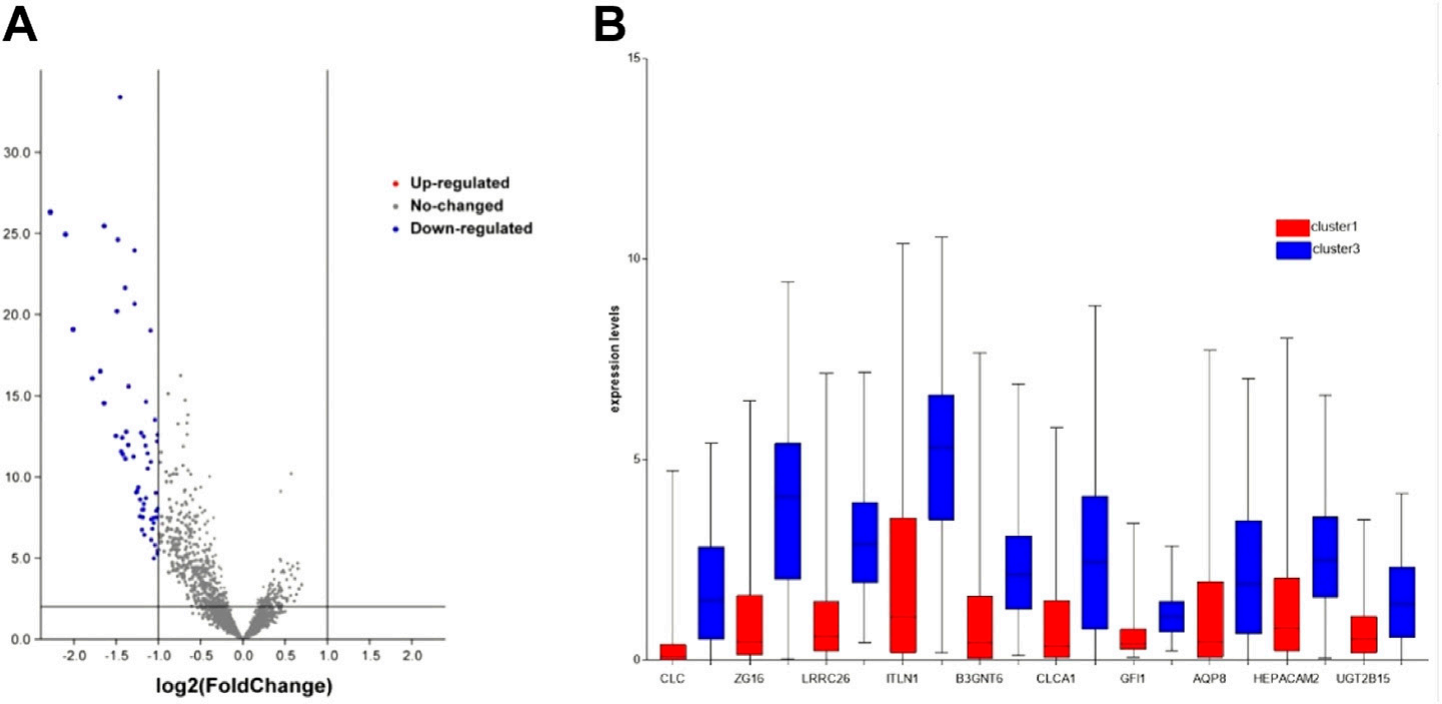
DEG’s expression between cluster1 and cluster3. **(A)** DEGs of two clusters *via* volcano. **(B)** DEGs with prognostic significance *via* boxplot.

**FIGURE 3:**
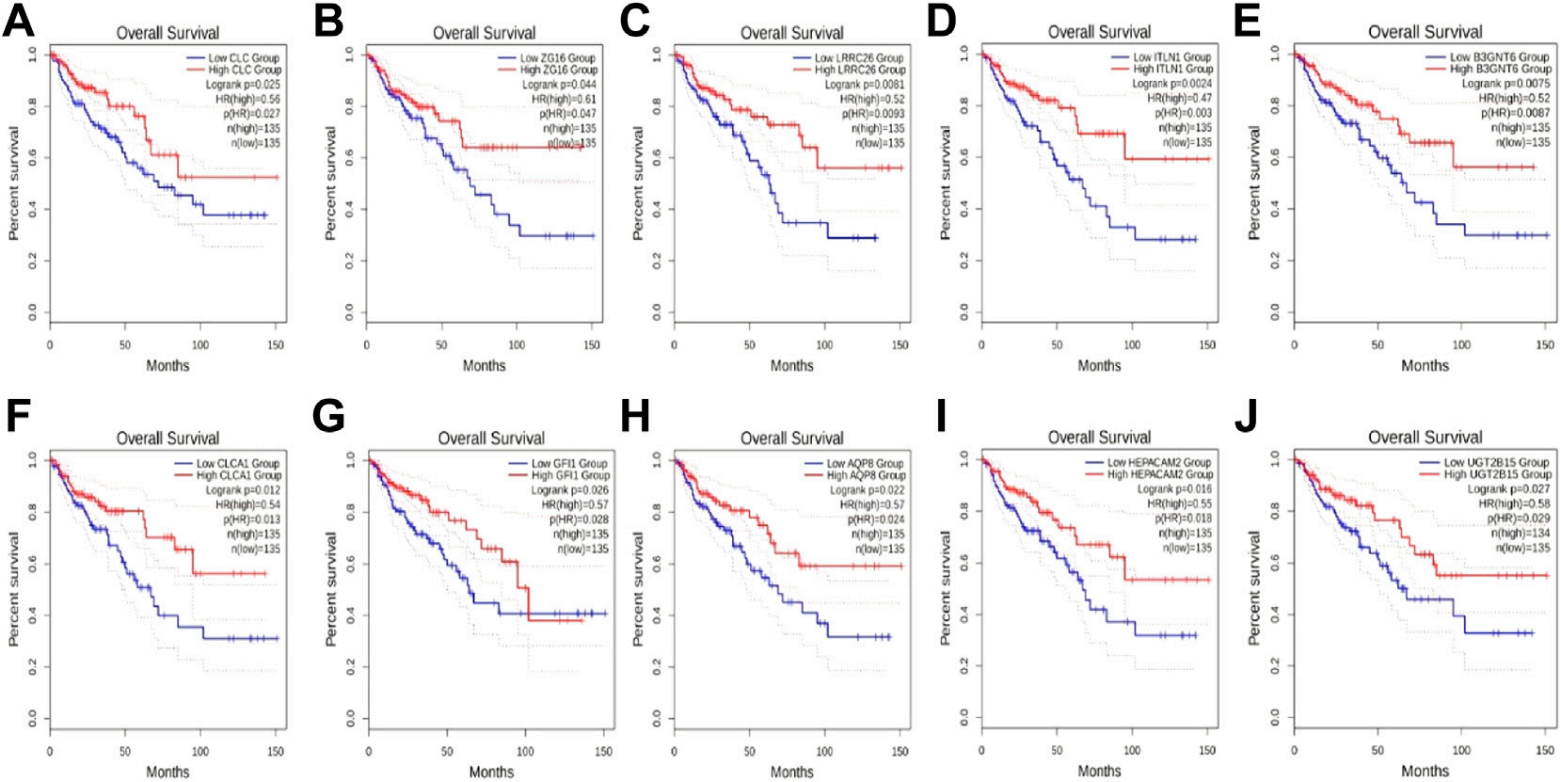
The effect of DEGs on survival time. **(A)** CLC, **(B)** ZG16, **(C)** LRRC26, **(D)** ITLN1, **(E)** B3GNT6, **(F)** CLCA1, **(G)** GFI1, **(H)** AQP8, **(I)** HEPACAM2, and **(J)** UGT2B15.

To explore which genes are responsible for the difference in survival times between cluster1 and cluster3, we performed a differential analysis of 5,000 genes in clusters 1 and 3 using package “limma”. A total of 58 genes were differentially expressed between the two clusters (Figure 2A). All DEGs were described in Supplementary Table S1. The expression levels of these genes in cluster3 were significantly higher than those in cluster1 (Figure 2B). Through external database validation with GEPIA2, we found a total of 10 genes whose differential expression played a significant role in prognosis, including CLC, ZG16, LRRC26, ITLN1, B3GNT6, CLCA1, GFI1, AQP8, HEPACAM2, and UGT2B15 (Figures 3A–J). The increased expression of these genes will have a better prognosis; when combined with the above model, we speculated that these genes will have a greater impact in CRC.

### Enrichment analysis

**FIGURE 4:**
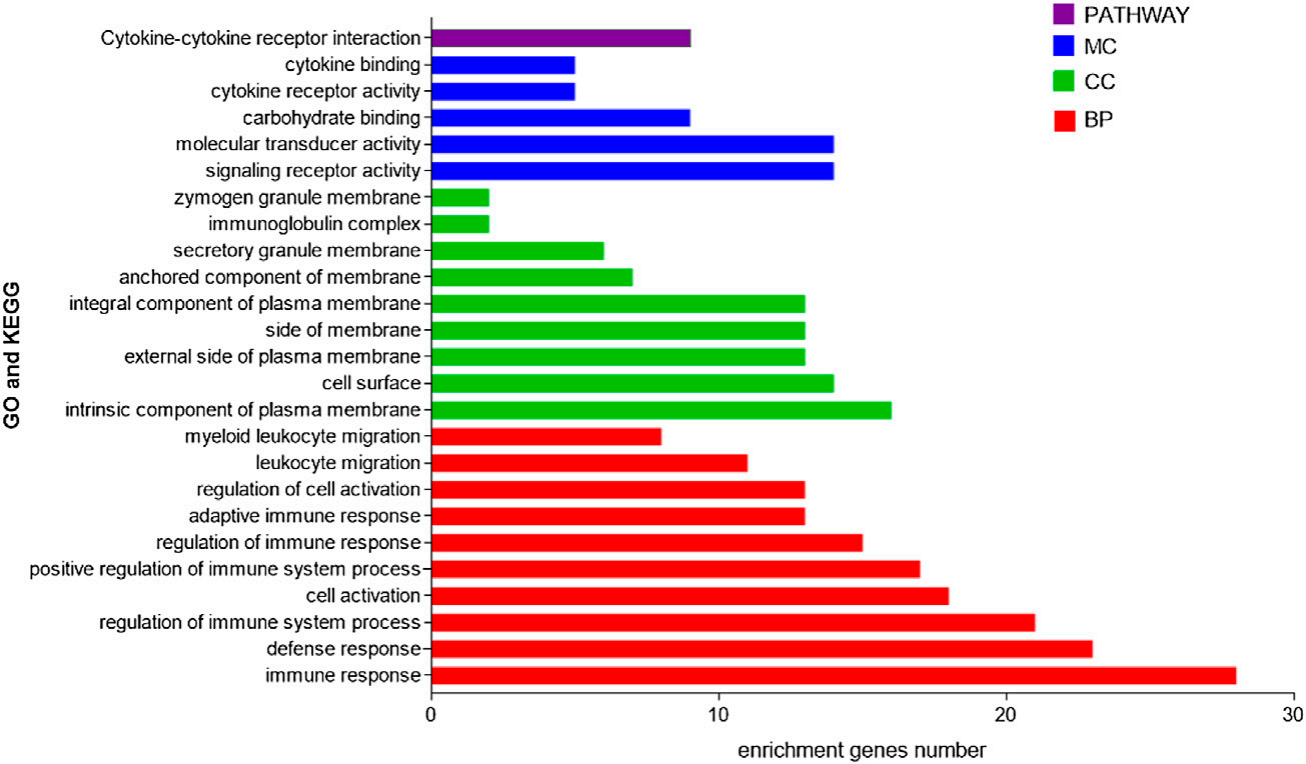
GO and KEGG enrichment analyses of DEGs in two subtypes.

We then used the WebGestalt online tool to perform enrichment analysis for all 58 DEGs between the two subtypes. Both GO and KEGG enrichment analysis results are shown in Figure 4, and the results showed that these genes were mainly enriched in immune-related pathways. GO includes immune response, defense response, and regulation of immune system processes. In addition, pathways related to cell morphology and cell membrane were also enriched, including cell activation, intrinsic component of the plasma membrane, and cell surface. The KEGG pathway showed that it is mainly enriched in the cytokine–cytokine receptor interaction pathway. These results suggest that immune factors play an important role in the prognosis of both subtypes.

### Genome alteration analysis

**FIGURE 5:**
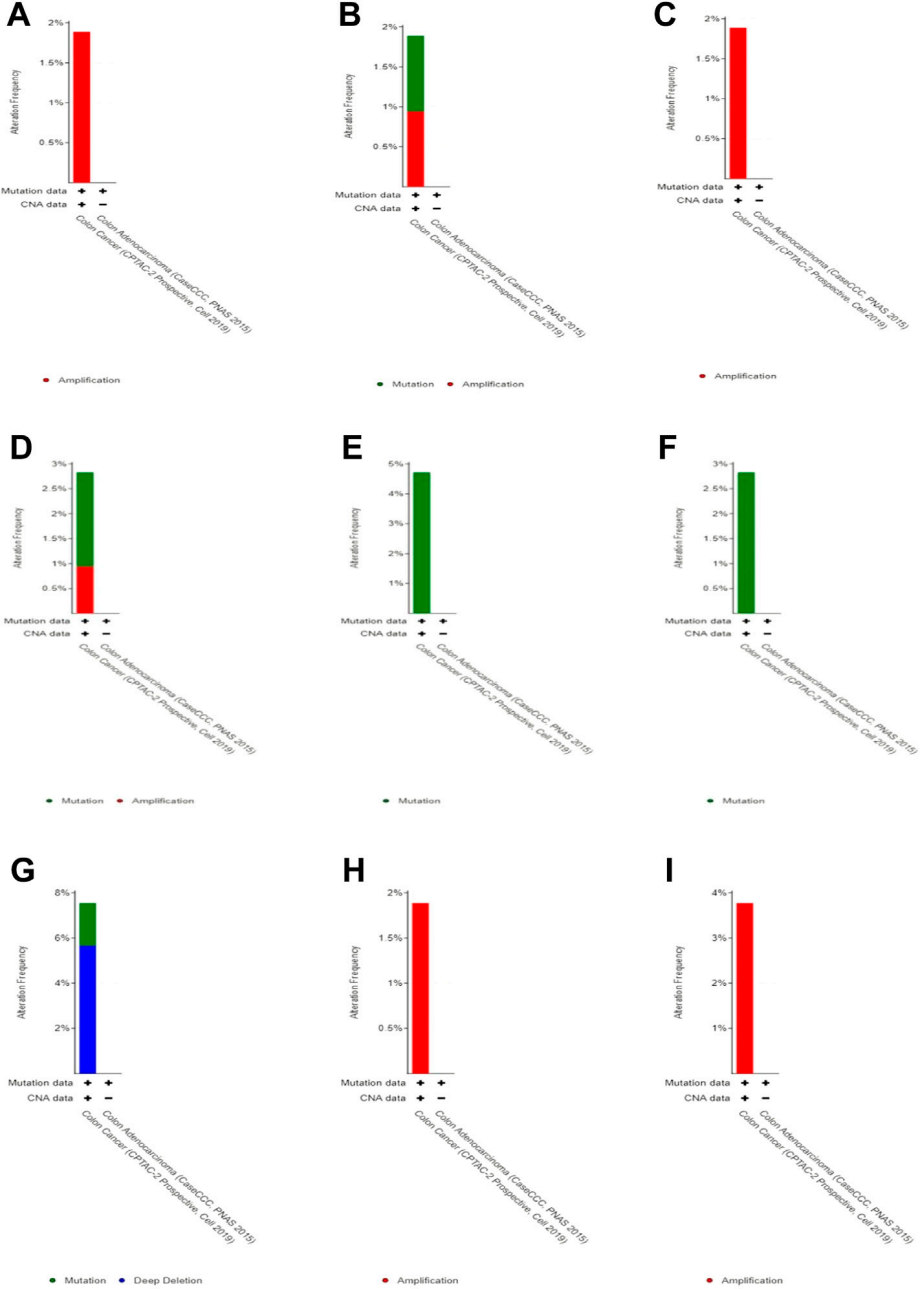
Genomic alteration in DEGs. **(A)** CLC, **(B)** ZG16, **(C)** LRRC26, **(D)** ITLN1, **(E)** B3GNT6,**(F)** CLCA1, **(G)** GFI1, **(H)** HEPACAM2, and **(I)** UGT2B15.

By studying changes in the genome, we found no significant changes (less than 5%) in each of these 10 genes (Figure 5). Therefore, we speculate that these changes do not have a significant impact on gene function.

### Protein–protein interaction network analysis

**FIGURE 6:**
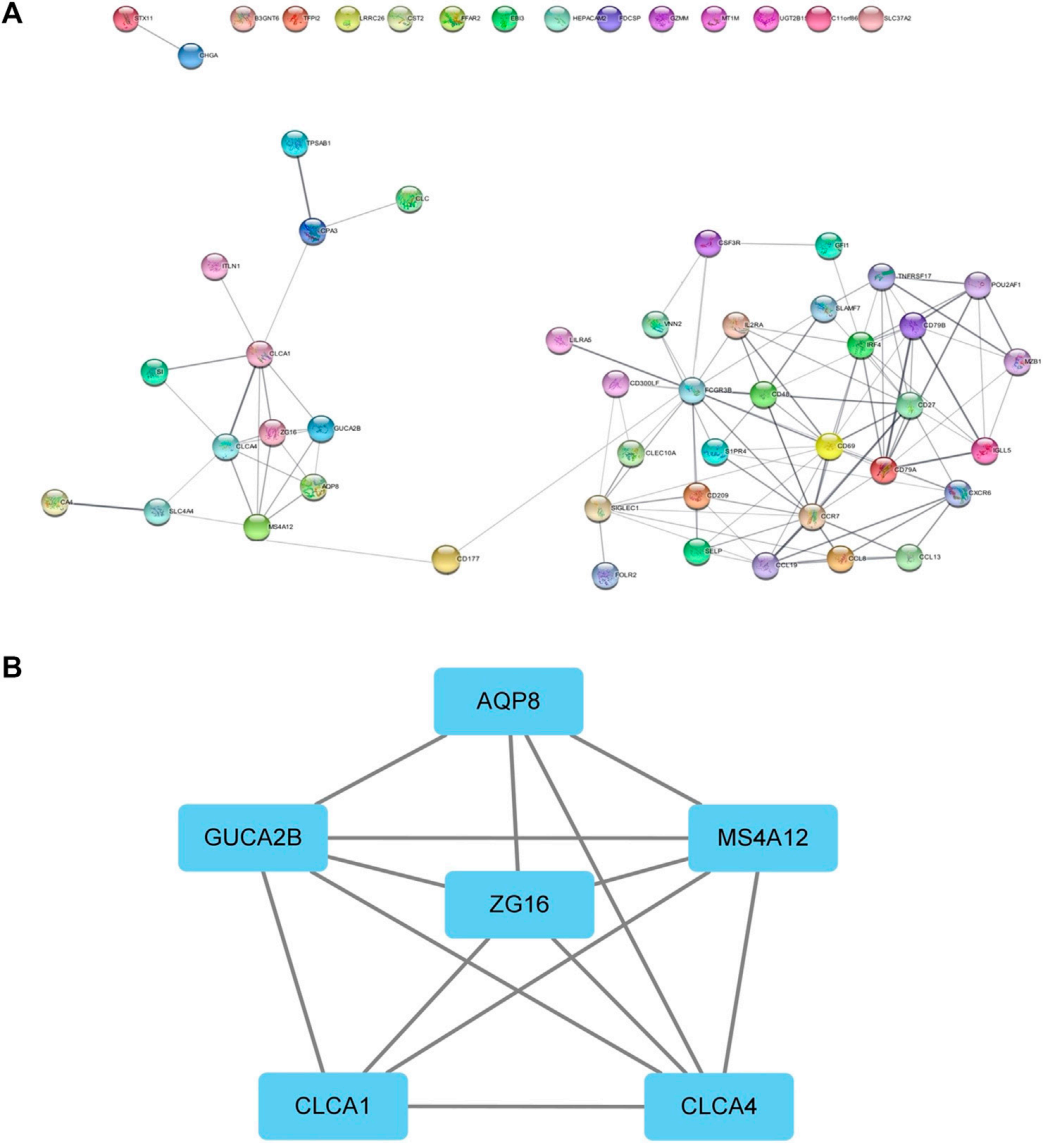
PPI network. **(A)** PPI of the STRING database. **(B)** Core regulatory networks selected by the MCODE plugin for Cytoscape.

We performed protein interaction analysis on DEGs using the STRING database (Figure 6A), and the results suggested that among the 10 genes with significant effects on CRC prognosis, CLC, ITLN1, ZG16, AQP8, CLCA1, and GFI1 interacted with other DEGs. This indicates that some genes play a role in the prognosis of CRC independently, and some of them may be regulated by other genes, thus having a complex impact on the prognosis of CRC. We used the MCODE plugin to select the core regulation network, setting the parameters as the degree cutoff of 2, node score cutoff of 0.2, and k-core of 2, and the module with an MCODE score >4 was presented. The results are shown in Figure 6B. These results suggest that AQP8 and ZG16 have an impact on the prognosis of CRC subtypes, but they are still regulated by other DEGs. The remaining DEGs have no direct impact on the prognosis of CRC subtypes, but they indirectly affect prognosis by regulating AQP8 and ZG16.

### Immune-infiltration analysis

**FIGURE 7:**
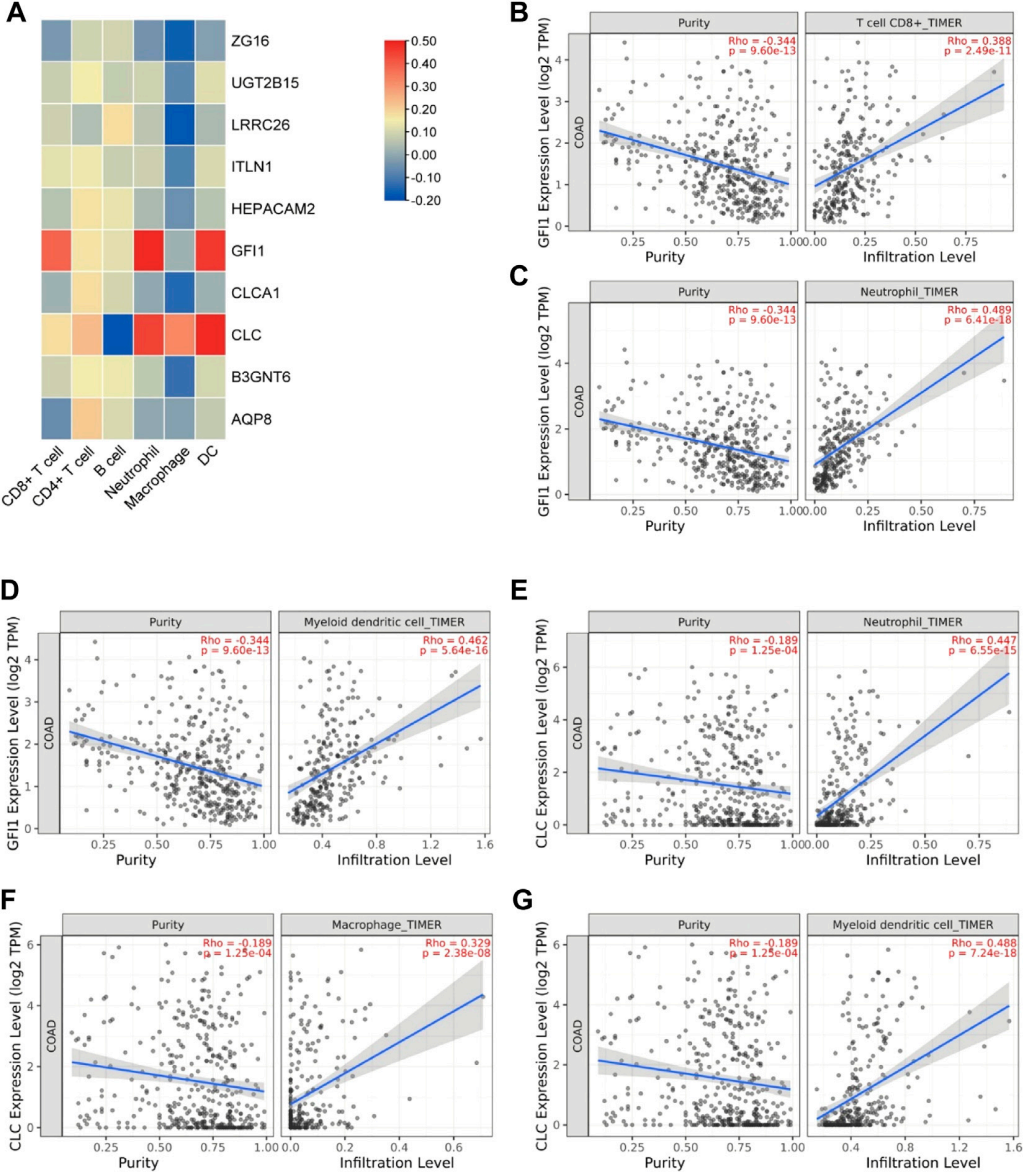
The relationship between DEGs and immune infiltrating cells. **(A)** Heatmap of the correlation between DEGs and immune cells. **(B)** Relationship between GFI1 and CD8^+^ T cells. **(C)** Relationship between GFI1 and neutrophils. **(D)** Relationship between GFI1 and DC. **(E)** Relationship between CLC and neutrophils. **(F)** Relationship between CLC and macrophages. **(G)** Relationship between CLC and DC.

**FIGURE 8:**
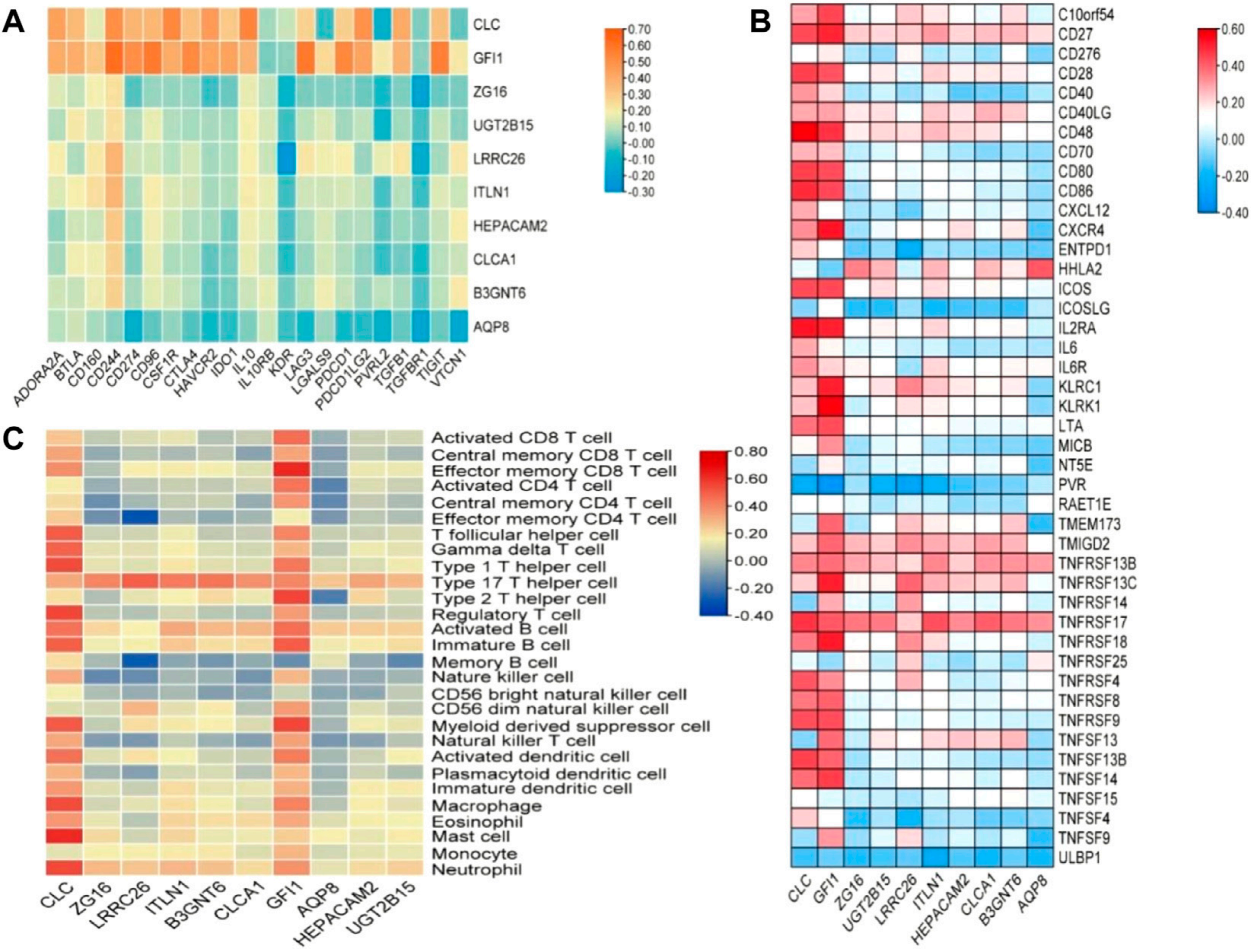
The relationship between DEGs and immunomodulator. **(A)** The relationship between DEGs and immunoinhibitor, **(B)** the relationship between DEGs and immunostimulator, and **(C)** relations between abundance of tumor-infiltrating lymphocytes (TILs) and expression of DEGs.

In order to explore the relationship between these 10 DEGs and immune genes and immune infiltrating cells, we used the TIMER2.0 database. As shown in Figure 7, GFI1 is significantly correlated with CD8^+^ T cells (cor = 0.388, *p* = 2.49e-11), neutrophils (cor = 0.489, *p* = 6.41e-18), and DC (cor = 0.462, *p* = 5.64e-16), and CLC is significantly correlated with DC (cor = 0.488, *p* = 7.24e-18), macrophage (cor = 0.329, *p* = 2.38e-8), and neutrophils (cor = 0.447, *p* = 6.55e-15). These results suggest that not only genes but also immune-infiltrating cells were involved in two subtypes of CRC. Then, we used the TISIDB database to explore the relationship between these 10 genes and immunoinhibitors and immunostimulators. CLC was significantly correlated with ADORA2A (cor = 0.444, *p* < 2.2e-16), CD244 (cor = 0.507, *p* < 2.2e-16), CSF1R (cor = 0.532, *p* < 2.2e-16), HAVCR2 (cor = 0.462, *p* < 2.2e-16), IL10 (cor = 0.544, *p* < 2.2e-16), PDCD1LG2 (cor = 0.492, *p* < 2.2e-16), and TGFB1 (cor = 0.41, *p* < 2.2e-16) of immunoinhibitor, and GFI1 was significantly correlated with ADORA2A (cor = 0.404, *p* < 2.2e-16), CD244 (cor = 0.623, *p* < 2.2e-16), CD274 (cor = 0.507, *p* < 2.2e-16), CD96 (cor = 0.581, *p* < 2.2e-16), CSF1R (cor = 0.407, *p* < 2.2e-16), CTLA4 (cor = 0.523, *p* < 2.2e-16), HAVCR2 (cor = 0.431, *p* < 2.2e-16), IDO1 (cor = 0.435, *p* < 2.2e-16), LAG3 (cor = 0.587, *p* < 2.2e-16), PDCD1 (cor = 0.529, *p* < 2.2e-16), PDCD1LG2 (cor = 0.436, *p* < 2.2e-16), and TIGIT (cor = 0.561, *p* < 2.2e-16) (Figure 8A). CLC was significantly correlated with CD27 (cor = 0.441, *p* < 2.2e-16), CD28 (cor = 0.457, *p* < 2.2e-16), CD48 (cor = 0.583, *p* < 2.2e-16), CD80 (cor = 0.458, *p* < 2.2e-16), CD86 (cor = 0.494, *p* < 2.2e-16), ICOS (cor = 0.441, *p* < 2.2e-16), IL2RA (cor = 0.534, *p* < 2.2e-16), TNFRSF17 (cor = 0.48, *p* < 2.2e-16), TNFRSF4 (cor = 0.423, *p* < 2.2e-16), TNFRSF9 (cor = 0.436, *p* < 2.2e-16), and TNFSF13B (cor = 0.457, *p* < 2.2e-16), and GFI1 was significantly correlated with C10orf54 (cor = 0.452, *p* < 2.2e-16), CD27 (cor = 0.506, *p* < 2.2e-16), CD28 (cor = 0.427, *p* < 2.2e-16), CD48 (cor = 0.483, *p* < 2.2e-16), CD80 (cor = 0.438, *p* < 2.2e-16), CD86 (cor = 0.444, *p* < 2.2e-16), CXCR4 (cor = 0.534, *p* < 2.2e-16), ICOS (cor = 0.444, *p* < 2.2e-16), IL2RA (cor = 0.514, *p* < 2.2e-16), KLRC1 (cor = 0.515, *p* < 2.2e-16), KLRK1 (cor = 0.572, *p* < 2.2e-16), LTA (cor = 0.44, *p* < 2.2e-16), TNFRSF13C (cor = 0.517, *p* < 2.2e-16), TNFRSF17 (cor = 0.417, *p* < 2.2e-16), TNFRSF18 (cor = 0.525, *p* < 2.2e-16), TNFRSF8 (cor = 0.403, *p* < 2.2e-16), TNFRSF9 (cor = 0.438, *p* < 2.2e-16), TNFSF13B (cor = 0.4, *p* < 2.2e-16), and TNFSF14 (cor = 0.465, *p* < 2.2e-16) of immunostimulator (Figure 8B). In the correlation of tumor-infiltrating lymphocytes (TILs) with DEGs, we found that CLC and GFI1 were significantly associated with immune cells. CLC was associated with T-follicular helper cells (Tfh, cor = 0.506, *p* < 2.2e-16), gamma delta T cells (Tgd, cor = 0.486, *p* < 2.2e-16), type 1 T-helper cells (Th1, cor = 0.544, *p* < 2.2e-16), regulatory T cells (Treg, cor = 0.555, *p* < 2.2e-16), activated B cells (Act_B, cor = 0.452, *p* < 2.2e-16), immature B cells (Imm_B, cor = 0.495, *p* < 2.2e-16), myeloid-derived suppressor cells (MDSC, cor = 0.509, *p* < 2.2e-16), activated dendritic cells (Act_DC, cor = 0.454, *p* < 2.2e-16), macrophages (cor = 0.545, *p* < 2.2e-16), mast cells (Mast, cor = 0.624, *p* < 2.2e-16), and neutrophils (cor = 0.548, *p* < 2.2e-16), and GFI1 was associated with activated CD8 T cells (Act_CD8, cor = 0.466, *p* < 2.2e-16), effector memory CD8 T cells (Tem_CD8, cor = 0.622, *p* < 2.2e-16), activated CD4 T cells (Act_CD4, cor = 0.448, *p* < 2.2e-16), Tfh (cor = 0.403, *p* < 2.2e-16), Th1 (cor = 0.441, *p* < 2.2e-16), type 2 T-helper cells (Th2, cor = 0.559, *p* < 2.2e-16), Act_B (cor = 0.463, *p* < 2.2e-16), Imm_B (cor = 0.501, *p* < 2.2e-16), MDSC (cor = 0.554, *p* < 2.2e-16), Act_DC (cor = 0.426, *p* < 2.2e-16), and macrophages (cor = 0.411, *p* < 2.2e-16) (Figure 8C). The results showed that GFI1 and CLC were significantly related to immune and inflammation factors, further suggesting that GFI1 and CLC may be involved in immune and inflammation factors in the process of regulating the prognosis of CRC subtypes.

## Discussion

With the development of technology, the scope of artificial intelligence in the medical field is also expanding. Unsupervised clustering is a technique that has been applied to the tumor level in recent years, and consensus clustering (CC) is used to estimate the number of unsupervised classes in a dataset, providing both quantitative and visual stability evidence (Wilkerson and Hayes, 2010; Greener et al., 2022). Using this technology to construct molecular subtypes of CRC can help us better understand the disease and develop new drugs and treatments. In this study, we constructed subtypes of CRC based on selected genes with significant prognostic differences. To investigate what caused this difference, we explored which of these genes is at work. The model we constructed found that cluster3 had a better prognosis than cluster1. When comparing the DEGs of two clusters, we found that there were 58 DEGs between the two clusters, and all of them were highly expressed in cluster3. Then, we used external databases to explore 10 genes (including CLC, ZG16, LRRC26, ITLN1, B3GNT6, CLCA1, GFI1, AQP8, HEPACAM2, and UGT2B15) that play a crucial role in the prognosis between the two clusters. The high expression of these 10 genes is associated with a better prognosis for CRC. Combined with the high expression of these genes in cluster3, the results also fit the conclusions from the model. Genomic analysis showed that the DEGs did not change significantly. Gene enrichment analysis provided a way for us to understand the functions of these genes. Through GO and KEGG enrichment analysis, we found that DEGs are closely related to immune function and inflammation. This suggests that the immune inflammation response system may play an important role in the prognosis of two clusters. Since these genes are highly expressed in cluster3, we hypothesized that the immune system has a positive effect on CRC in cluster3 and these DEGs interact with the immune system to improve prognosis. To test our hypothesis, we analyzed 10 DEGs associated with the immune system. Results show that CLC and GFI1 have the highest correlation with immunity. The immunosuppressive pathway can regulate the immune environment of the body and prevent the over-activation of the immune mechanism from causing damage to itself, and immunoinhibitors are the key factors in this regulation. Immune stimulation is to activate the immune system, enhance our immunity, and play an important role in responding to foreign pathogens, removing self-damaging cells and monitoring and inhibiting the occurrence and development of tumors. Our study suggests that CLC and GFI1 have dual roles in regulating immune mechanisms and resulting in a better prognosis for CRC. The protein encoded by CLC is a lysophospholipase that is expressed on eosinophils and basophils, implying its function in relation to inflammation (Su, 2018).

Next, it was found through enrichment analysis that the DEGs of the two CRC subtypes were related to the inflammatory pathway, and the PPI network revealed that these genes interacted with CLC, indicating that the inflammatory response has an important role in CRC subtypes in prognosis. Previous research found that CLC can activate macrophages to secrete IL-1β, thereby aggravating inflammation (Rodriguez-Alcazar et al., 2019). Our study also came to the same conclusion. CLC has a significant correlation with macrophages and mast cells, so inflammatory mediators under the regulation of CLC in CRC play a role in the prognosis of both subtypes. Future studies are expected to explore the role and mechanism of the inflammatory response. GFI1 encodes a nuclear zinc finger protein, which functions as a transcriptional repressor. It has also been shown to be associated with neutrophils (the TIMER database also shows a correlation). When GFI1 is mutated, it can lead to neutropenia (Moroy et al., 2015). The relationship between GFI1 and immunoinhibitors and immunostimulators, as well as the way of regulating TILs, provides a direction for the development of new targeted drugs in the future. In addition to the impact of immune-inflammatory factors on the prognosis of both subtypes, ZG16, ITLN1, CLCA1, AQP8 and other genes encoding the transport channels on the cell membrane and involved in the transport of intracellular substances are also among the prognostic factors. Although the remaining DEGs did not directly affect the prognosis of the two subtypes of CRC, we found that by constructing the protein interaction network of all DEGs, they could interact with CLC, GFI1, ZG16, ITLN1, CLCA1, and AQP8 and indirectly participate in the regulation of prognosis of the subtypes of CRC. According to the model, we found that the factors that cause the difference in the prognosis of CRC are very complex, which is the result of multiple factors.

In summary, our study revealed the prognostic factors affecting CRC based on immunity, inflammation, transporters, and ion channels. Despite the positive results, this study has a number of limitations. For one thing, due to the differences in the original data and algorithms of the database, the results of this study may be biased. The small sample size of the database may also lead to discrepancies in the data, and real-time updates to the database can also change results. For another, our data need to be confirmed by *in vivo*/*in vitro* experiments, such as gene expression or proteomic analyses based on clinical samples. In the future, research should further explore the mechanism of action and pathogenesis of these genes in order to validate the proposed model’s effectiveness and provide a new way for the treatment of CRC.

## Data Availability

The datasets presented in this study can be found in online repositories. The names of the repository/repositories and accession number(s) can be found in the article/Supplementary Material.
