# Supplementary material for: Constructing a molecular subtype model of colon cancer using machine learning
Source: Front Pharmacol. 2022 Sep 16;13:1008207. doi: 10.3389/fphar.2022.1008207 (PMC9523145; doi:10.3389/fphar.2022.1008207)
Supplement: Supplementary file 1 [file Table1.docx]

**Table S1.** **Significant differential genes between cluster 1 and cluster 3**.

| Gene | log2FoldChange | p-value |
| --- | --- | --- |
| CLC | -2.278834319 | 4.83E-27 |
| TNFRSF17 | -2.097825027 | 1.15E-25 |
| GUCA2B | -2.009351929 | 8.29E-20 |
| IRF4 | -1.781093603 | 8.50E-17 |
| CCL13 | -1.686313303 | 3.05E-17 |
| CLEC10A | -1.642474851 | 2.90E-15 |
| ZG16 | -1.641301162 | 3.44E-26 |
| POU2AF1 | -1.501994106 | 2.96E-13 |
| CA4 | -1.490349484 | 6.32E-21 |
| LRRC26 | -1.480196784 | 2.41E-25 |
| IGLL5 | -1.451969795 | 3.88E-34 |
| GZMM | -1.439582427 | 2.74E-12 |
| CD177 | -1.427907354 | 3.82E-13 |
| CD209 | -1.421338302 | 4.10E-12 |
| CD79A | -1.392709397 | 2.28E-22 |
| CD79B | -1.389251786 | 7.60E-12 |
| CLCA4 | -1.380339461 | 1.65E-13 |
| CHGA | -1.357082259 | 1.08E-12 |
| SLAMF7 | -1.35256694 | 2.61E-16 |
| MS4A12 | -1.294391911 | 5.66E-12 |
| ITLN1 | -1.281095808 | 1.11E-24 |
| MZB1 | -1.28028538 | 2.17E-21 |
| STX11 | -1.260865293 | 9.17E-10 |
| SLC37A2 | -1.252726289 | 7.69E-10 |
| EBI3 | -1.236279183 | 4.44E-10 |
| TFPI2 | -1.217385231 | 2.74E-08 |
| CD300LF | -1.216120836 | 2.50E-09 |
| C11orf86 | -1.205017804 | 1.94E-13 |
| FCGR3B | -1.194231286 | 1.87E-07 |
| CCR7 | -1.191412307 | 1.05E-08 |
| FFAR2 | -1.184610876 | 3.13E-08 |
| IL2RA | -1.174250981 | 1.01E-08 |
| B3GNT6 | -1.172415364 | 3.24E-13 |
| SLC4A4 | -1.17164903 | 4.61E-09 |
| VNN2 | -1.165239166 | 3.79E-07 |
| MT1M | -1.151317514 | 1.19E-12 |
| SI | -1.146619432 | 2.02E-09 |
| CPA3 | -1.146026186 | 2.32E-15 |
| CD27 | -1.127026868 | 3.58E-12 |
| CCL19 | -1.125693038 | 3.09E-11 |
| CLCA1 | -1.092742728 | 9.66E-20 |
| CD48 | -1.089821906 | 1.18E-11 |
| LILRA5 | -1.085655342 | 4.03E-08 |
| CCL8 | -1.0846254 | 7.65E-07 |
| CD69 | -1.07147117 | 1.60E-07 |
| CXCR6 | -1.059658847 | 3.49E-08 |
| GFI1 | -1.057329834 | 7.04E-08 |
| FDCSP | -1.054913863 | 1.05E-05 |
| TPSAB1 | -1.041931352 | 3.05E-14 |
| SELP | -1.041599374 | 1.61E-06 |
| CST2 | -1.031118981 | 9.85E-10 |
| AQP8 | -1.026765964 | 3.20E-08 |
| S1PR4 | -1.026270146 | 1.27E-08 |
| FOLR2 | -1.016233895 | 6.46E-13 |
| HEPACAM2 | -1.010203644 | 2.50E-13 |
| SIGLEC1 | -1.010112393 | 5.56E-06 |
| CSF3R | -1.009665811 | 3.52E-06 |
| UGT2B15 | -1.008043742 | 9.29E-09 |
