# Supplementary material for: Constructing a molecular subtype model of colon cancer using machine learning
Source: Front Pharmacol. 2022 Sep 16;13:1008207. doi: 10.3389/fphar.2022.1008207 (PMC9523145; doi:10.3389/fphar.2022.1008207)
Supplement: Supplementary file 2 [file Image1.pdf]

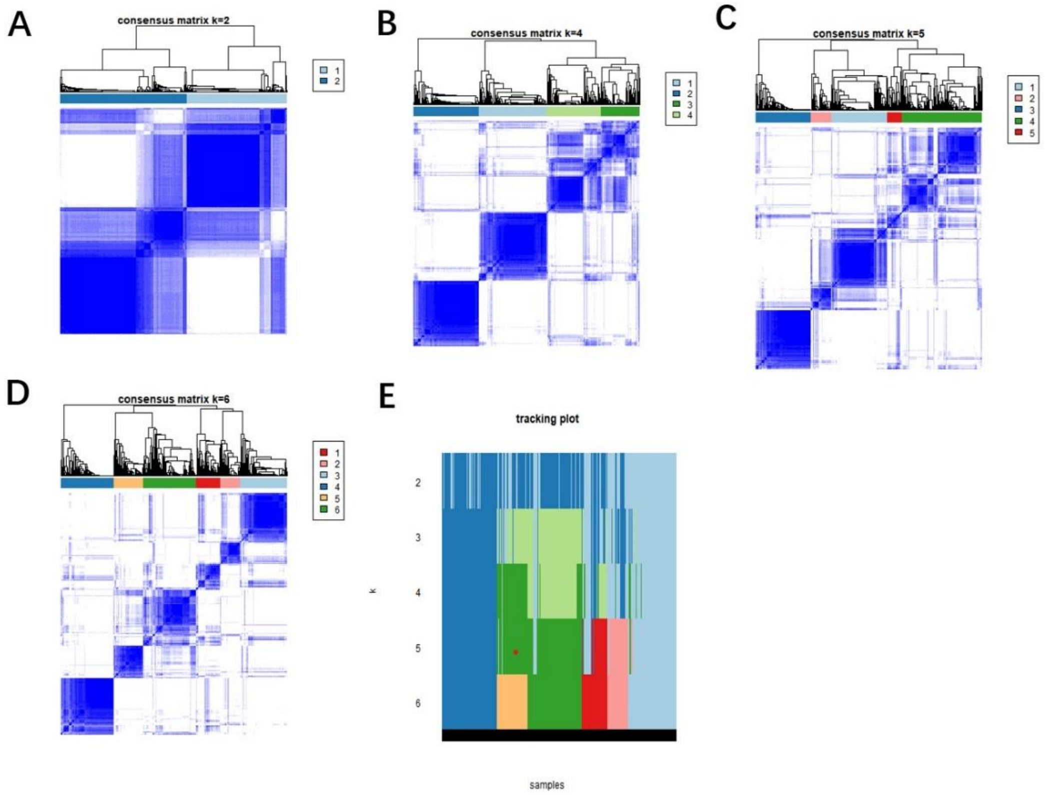

Supplementary Figure S1. The rest of the clustering results. (A) k=2, consensus clustering analysis yields 2 matrices, each representing 1 colon cancer subtype. (B) k=4, consensus clustering analysis yields 4 matrices, each representing 1 colon cancer subtype.(C) k=5,consensus clustering analysis yields 5 matrices, each representing 1 colon cancer subtype.(D) k=6,consensus clustering analysis yields 6 matrices, each representing 1 colon cancer subtype.(E) Display the clustering results in the form of a tracking plot.
